# The origin of *Wx* ^*la*^ provides new insights into the improvement of grain quality in rice

**DOI:** 10.1111/jipb.13011

**Published:** 2021-03-26

**Authors:** Hao Zhou, Duo Xia, Da Zhao, Yanhua Li, Pingbo Li, Bian Wu, Guanjun Gao, Qinglu Zhang, Gongwei Wang, Jinghua Xiao, Xianghua Li, Sibin Yu, Xingming Lian, Yuqing He

**Affiliations:** ^1^ National Key Laboratory of Crop Genetic Improvement and National Centre of Plant Gene Research (Wuhan) Huazhong Agricultural University Wuhan 430070 China

**Keywords:** allelic variation, appearance and eating quality, intragenic recombination, *Oryza sativa*, *waxy*

## Abstract

Appearance and taste are important factors in rice (*Oryza sativa*) grain quality. Here, we investigated the taste scores and related eating‐quality traits of 533 diverse cultivars to assess the relationships between—and genetic basis of—rice taste and eating‐quality. A genome‐wide association study highlighted the *Wx* gene as the major factor underlying variation in taste and eating quality. Notably, a novel *waxy* (*Wx*) allele, *Wx* ^*la*^, which combined two mutations from *Wx* ^*b*^ and *Wx* ^*in*^, exhibited a unique phenotype. Reduced GBSSI activity conferred *Wx* ^*la*^ rice with both a transparent appearance and good eating quality. Haplotype analysis revealed that *Wx* ^*la*^ was derived from intragenic recombination. In fact, the recombination rate at the *Wx* locus was estimated to be 3.34 kb/cM, which was about 75‐fold higher than the genome‐wide mean, indicating that intragenic recombination is a major force driving diversity at the *Wx* locus. Based on our results, we propose a new network for *Wx* evolution, noting that new *Wx* alleles could easily be generated by crossing genotypes with different *Wx* alleles. This study thus provides insights into the evolution of the *Wx* locus and facilitates molecular breeding for quality in rice.

## Graphical abstract

*Wx^la^* is derived from intragenic recombination of the *Wx^b^* and *Wx^in^* alleles, and *Wx^la^* rice grains have a transparent appearance and excellent eating quality. The study of *Wx^la^* provides insight on the evolution of the *Wx* gene and suggests avenues for improvement of rice grain quality.

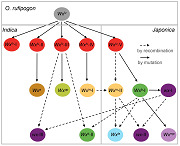

## INTRODUCTION

Rice is the staple food for more than one‐third of the world's population. As a result of economic development and improvements in living standards, rice quality has become a major factor in rice production, due to its effect on market value and farmer incomes (Zhang, 2007; Zhang et al., 2008). Scientists in China have proposed the concept of Super Green Rice, with the objective of developing environmentally friendly rice varieties with high yield and good quality (Zhang, 2007). Processed white rice is the main form of rice as a commodity, and appearance and taste are the most important traits demanded by consumers (Zhou et al., 2019). Appearance is determined mainly by grain shape and transparency, whereas eating quality comprises physicochemical properties that include apparent amylose content (AAC), gel consistency (GC), gelatinization temperature (GT), and viscosity. Clarification of the relationships between quality traits and taste and elucidation of their genetic basis are main objectives in meeting the breeding goals for Super Green Rice.

Many studies in rice have aimed to dissect the genetic underpinnings of eating quality. The *Waxy* (*Wx*) gene, which encodes granule‐bound starch synthase I (GBSSI) and controls amylose content in the endosperm, is a major determinant of eating quality (Traore et al., 2011). In glutinous (*waxy*) rice, a 23‐bp repeat in the second exon of *Wx* causes the premature termination of translation and loss of GBSSI function (Wanchana et al., 2003). Several *Wx* alleles confer diverse GBSSI activities that affect amylose content (Ayres et al., 1997; Larkin and Park, 2003; Mikami et al., 2008; Zhang et al., 2019). *Wx* ^*lv*^ was reported to be the ancestral allele of the *Wx* gene in rice and the three main *Wx* alleles (*Wx* ^*a*^ *, Wx* ^*b*^ *, Wx* ^*in*^) differ from *Wx* ^*lv*^ by substitution of functional residues (Zhang et al., 2019). Gelatinization temperature is mainly controlled by *ALK*, which encodes a putative soluble starch synthase II (SSIIa) and positively regulates GT (Gao et al., 2003). Most *indica* varieties have a high GT, caused by enhanced activity of *ALK*, which increases the contents of medium and long amylopectin chains that are more difficult to gelatinize. In addition to *Wx* and *ALK*, several genes that have been identified via mutants are thought to influence eating quality. For example, the *flo5* mutant generated from a T‐DNA insertion in *SSIIIa*, which controls amylopectin chain elongation, shows a degree of polymerization ≥30 and a floury endosperm phenotype with decreased viscosity (Fujita et al., 2007). Moreover, mutation of *starch‐branching enzyme I* (*SBEI*) decreases GT, leading to improved eating quality (Satoh et al., 2003).

Despite these studies, the relationships between taste and grain‐quality traits, and the genetic basis of taste, remain unclear. Sensory testing is a direct way to evaluate taste in rice, but it is not practical for use in breeding or research, due to limitations in the numbers of professional tasters and the large amounts of cooked rice required for such tests (Lyon et al., 1999; Limpawattana and Shewfelt, 2010; Kwak et al., 2015). However, a commercially available taste analyzer can accurately predict taste and is suitable for scientific research (Champagne et al., 1996). In this study, we used taste scores (TSs) obtained from a rice taste analyzer (see *Methods*) to study the relationship between taste and eating‐quality traits in 533 different rice varieties. We performed genome‐wide studies on TS and eating‐quality traits to identify genes that are important for eating quality. Our results provide insights into the genetic bases of rice quality and have new genetic resource for rice breeding.

## RESULTS

### Phenotyping reveals eating‐quality trait relationships

**Figure 1:**
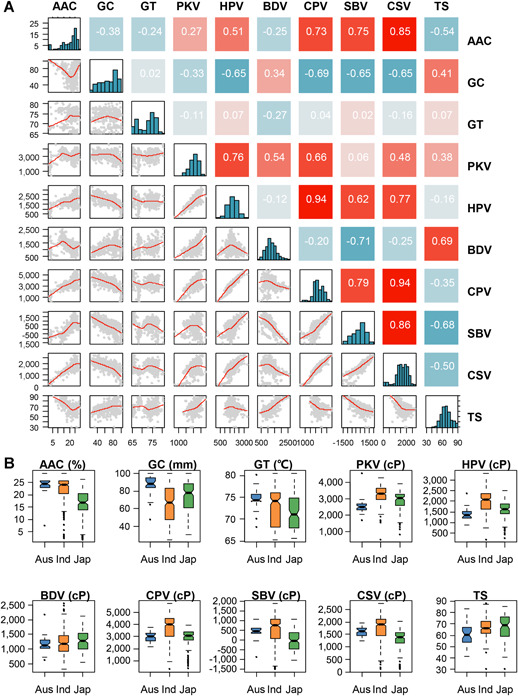
Phenotypic analysis reveals trait relationships and subpopulation characteristics (**A**) Heatmap depicting Pearson's correlation coefficients between phenotype means for quality traits across all varieties within the study. Phenotypic distributions of quality traits are located within the diagonal. (**B**) Phenotypic distributions of quality traits divided by the *aus* (Aus), *indica* (Ind), and *japonica* (Jap) subpopulations. The number of varieties within each subpopulation was 50, 307, and 176, respectively. cP, centipoise.

To clarify the relationship between TS and eating‐quality traits, we investigated AAC, GC, GT, viscosity characteristics, and the TS in 533 different rice cultivars (Figure 1A). The viscosity characteristics obtained from a rapid visco‐analyzer (RVA) included six parameters (Figure S1); namely, peak viscosity (PKV), hot paste viscosity (HPV), breakdown viscosity (BDV), cold paste viscosity (CPV), setback viscosity (SBV), and consistency viscosity (CSV). The TS ranged from 30 (less preferred) to 87.8 (more preferred) in non‐glutinous varieties. The TS was positively correlated with GC, PKV and BDV and negatively correlated with AAC, SBV, and CSV (Figure 1). Among these quality traits, BDV and SBV contributed most to the TS.

Subpopulation differences are well known in *O. sativa*, especially between subspecies *indica* and *japonica* (Huang et al., 2012). Eating‐quality traits also showed distinct distributions within subpopulations (Figure 1B). In a previous study, a collection of 533 different cultivated rice accessions was divided into *aus*, *indica* and *japonica* groups, containing 50, 305, and 178 accessions, respectively (Zhou et al., 2017). The *indica* group had a higher AAC than the *japonica* group, as a result of different distributions of known *Wx* alleles (Ayres et al., 1997). The *japonica* group showed a higher TS than the *indica* group, and a correspondingly higher BDV and lower SBV. These results demonstrated the diversity among the accessions.

### *Wx* is the main gene that confers eating quality

**Figure 2:**
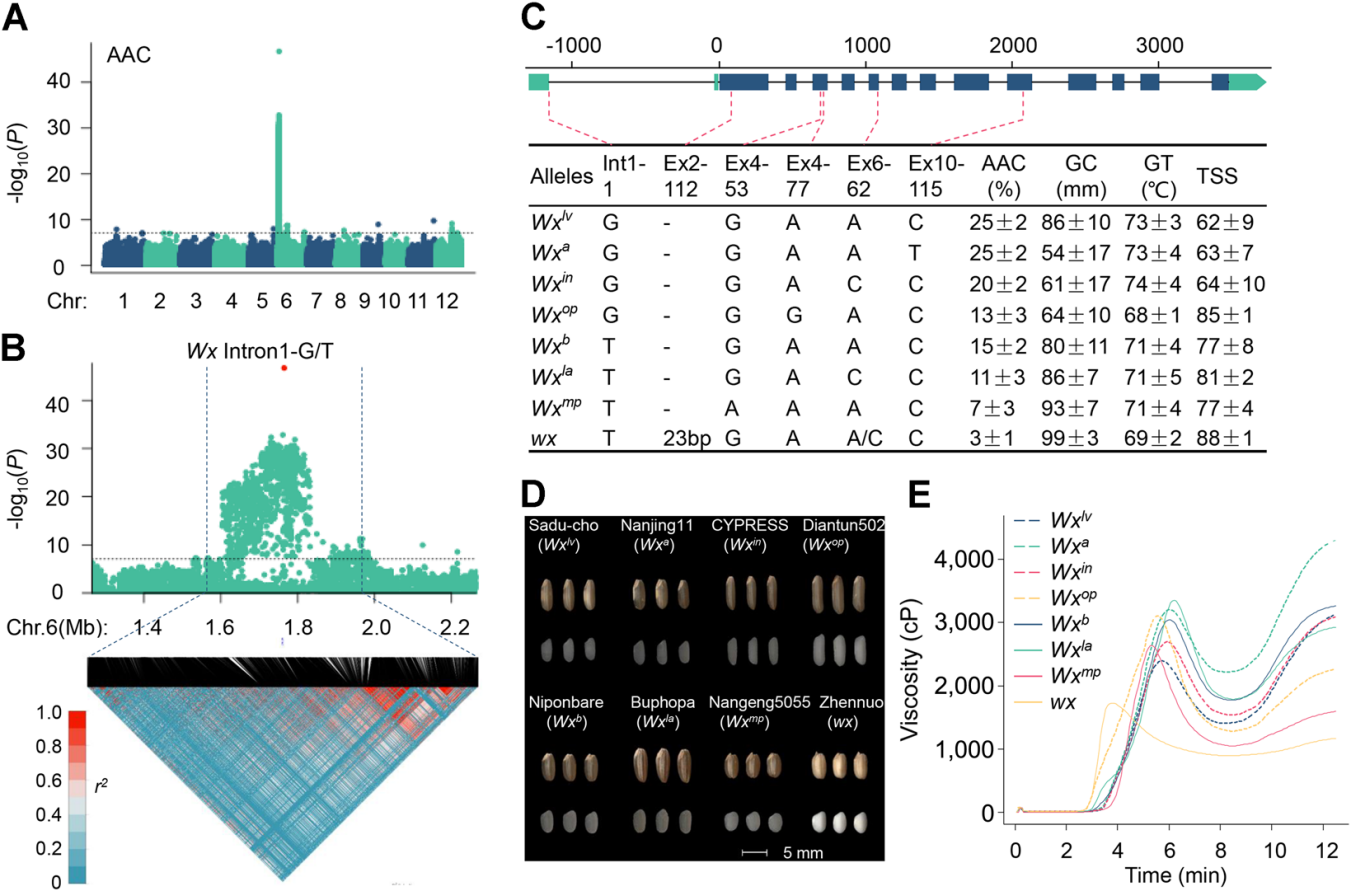
Association study identification of multiple *Wx* alleles conferring different qualities (**A**) Manhattan plot for apparent amylose content (AAC) in the whole population. The dashed line represents the significance threshold (−log_10_(*P*) = 7.18). (**B**) Local Manhattan plot (top) and LD heatmap (bottom) surrounding the peak on chromosome 6. The red dot indicates the position of nucleotide variation in the candidate gene. (**C**) Genotypes and phenotypes of different *Wx* alleles in 533 different rice varieties. (**D**) The appearance of brown rice and milled rice for representative cultivars carrying different *Wx* alleles. (**E**) Representative rapid visco‐analyzer (RVA) pasting properties for different *Wx* alleles. cP, centipoise.

We performed a genome‐wide association study for nine eating‐quality traits and obtained TSs for the entire group of accessions. The *Wx* gene was identified as a major gene affecting AAC, GC, all RVA characteristics, and TS, whereas *ALK* was identified to influence GT (Figure S2; Table S1). Because no strong LD blocks were observed around the *Wx* locus, the peak SNP on chromosome 6 was identified to be the major functional site underlying the observed variation in *Wx* (Figure 2A, B). The G‐to‐T SNP in intron 1 divided cultivated rice into high AAC and intermediate AAC genotypes, and this variation explained 60.02% of the phenotypic variance in AAC and 33.63% of the variation in TS (Table S1). A stepwise regression for all *Wx* variants identified five known functional variants (Table S2). The four major variants, Int1‐1, Ex2‐112, Ex4‐77, and Ex6‐62, explained up to 80.64% of the phenotypic variance in AAC. We then divided the *Wx* locus into eight alleles according to these six variations (Figure 2C). Different *Wx* alleles conferred different AAC, GC, TS, appearance, and RVA characteristics (Figure 2C–E).

### A new *Wx* allele, *Wx* ^*la*^

Seven out of the eight *Wx* alleles have been previously described, but one allele displayed a low AAC, a high GC, good RVA characteristics, and an attractive TS. Because this allele exhibited a low AAC and a transparent appearance, we named it *Wx* ^*la*^ (Figure 2C, D). The *Wx* ^*b*^ allele contained the Int1‐1 mutation, the *Wx* ^*in*^ allele contained the Ex6‐62 mutation, and *Wx* ^*la*^ contained a combination of both mutations. The *Wx* ^*op*^ and *Wx* ^*mp*^ alleles also exhibited a low AAC but an opaque appearance (Figure 2D). Single SNP differences existed between any two combinations of *Wx* ^*b*^, *Wx* ^*la*^ and *Wx* ^*mp*^, but quality assessments of varieties containing each allele differed. The AAC, GC and TS of Bophopa, which carried the *Wx* ^*la*^ allele, were intermediate between those of Nipponbare and Nangeng5055, which carried the *Wx* ^*b*^ and *Wx* ^*mp*^ alleles, respectively. Previous studies of diverse rice collections indicated that the Int1‐1 and Ex6‐62 variants at the *Wx* locus were associated with a low AAC (Larkin and Park, 2003; Hoai et al., 2014). However, the effect of this haplotype on rice grain quality was not analyzed.

### *Wx* ^*la*^ exhibits an intermediate genetic effect between that of *Wx* ^*b*^ and *Wx* ^*mp*^

**Figure 3:**
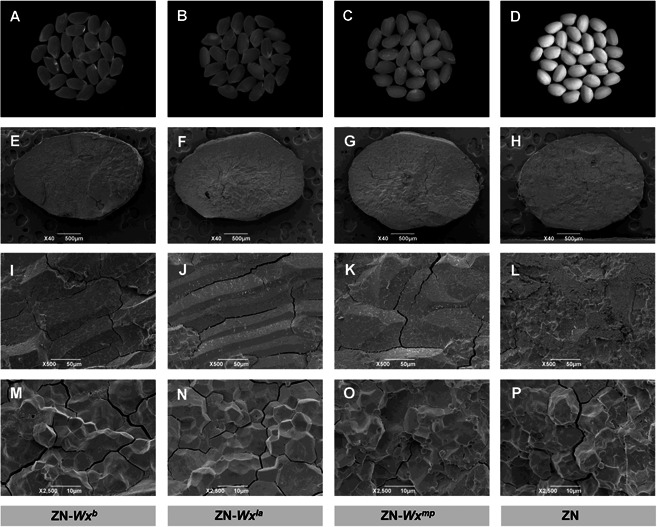
The appearance and morphology of starch granules for different *Wx* alleles of white rice (**A–D**) Milled rice from genotypes ZN‐*Wx* ^*b*^ (**A**), ZN‐*Wx* ^*la*^ (**B**), ZN‐*Wx* ^*mp*^ (**C**) and ZN (**D**). **(E–P**) SEM images showing the morphology of the starch granules of ZN‐*Wx* ^*b*^ (**E, I, M**), ZN‐*Wx* ^*la*^ (**F, J, N**), ZN‐*Wx* ^*mp*^ (**G, K, O**) and ZN (**H, L, P**). Resolution, 40× (**E–H**), 500X (**I–L**) and 2,500X (**M–P**).

**Figure 4:**
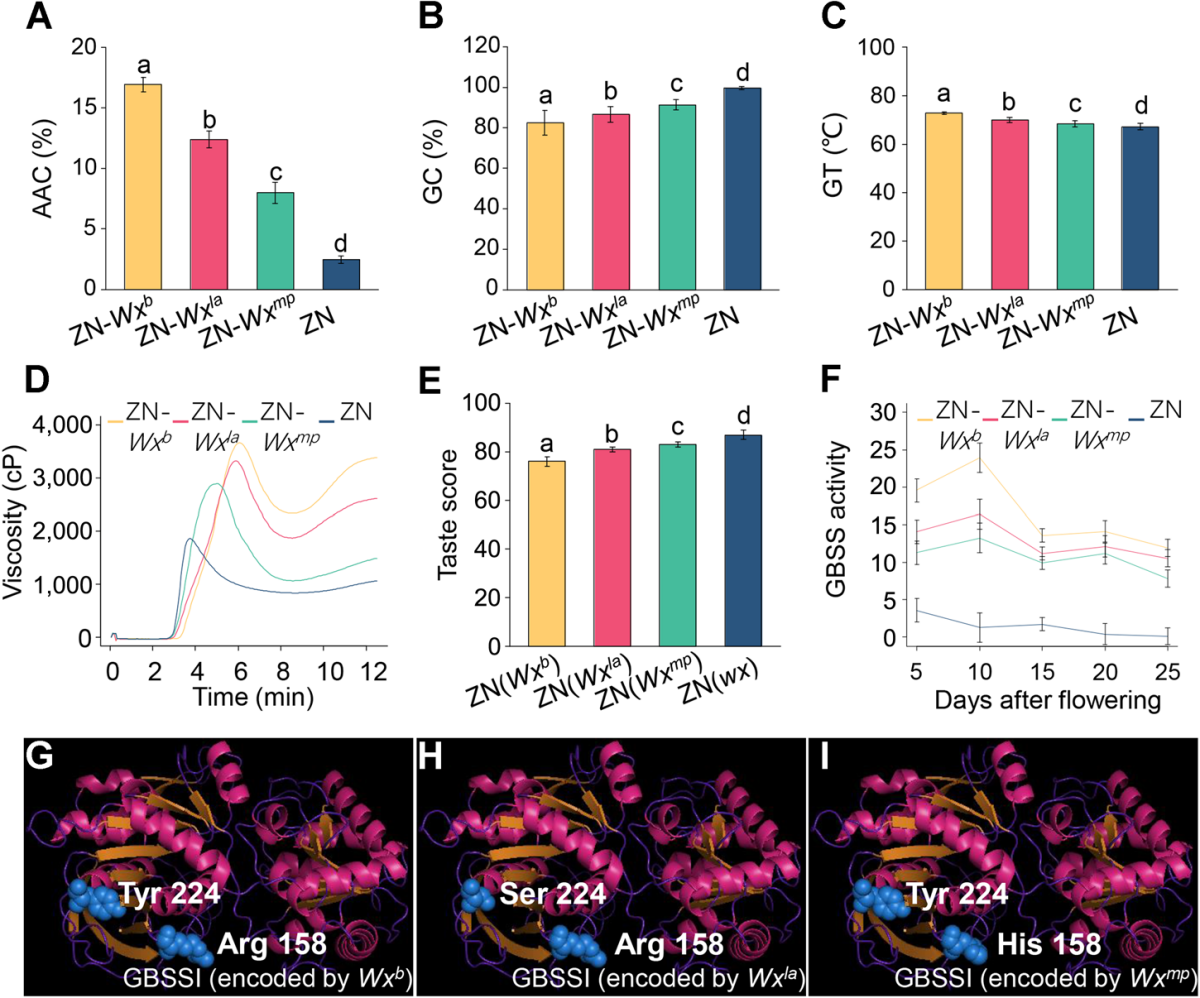
Different GBSSI enzyme activities lead to differences in qualities conferred by *Wx*^*b*^, *Wx*^*la*^ and *Wx*^*mp*^ (**A–E**) The apparent amylose content (AAC) (**A**), gel consistency (GC) (**B**), gelatinization temperature (GT) (**C**), rapid visco‐analyzer (RVA) pasting properties (**D**) and taste score (**E**) of *Wx* ^*b*^, *Wx* ^*la*^ and *Wx* ^*mp*^ in the ZN background. (**F**) The GBSSI activity of different *Wx* alleles during endosperm development. (**G**–**I**) Three‐dimensional homology modeling of GBSSI encoded by *Wx* ^*b*^ (**G**), *Wx* ^*la*^ (**H**) and *Wx* ^*mp*^ (**I**). ZN is a glutinous rice variety containing the *wx* allele. Error bars represent the SD of means (*n* = 5). Different letters above the bars indicate significant differences at *P* < 0.05, using Tukey's multiple‐comparison test. cP, centipoise.

To investigate further the relationship between DNA sequence variation at the *Wx* locus and eating quality and to understand how the *Wx* alleles regulate eating quality at the physiological level, the genetic effect of *Wx* alleles, including quality traits and GBSS activity, required evaluation in the same genetic background. We transformed alleles *Wx* ^*b*^, *Wx* ^*la*^ and *Wx* ^*mp*^ into glutinous *japonica* variety Zhennuo (ZN), which carries the nonfunctional *Wx* allele, *wx*. These *Wx* alleles conferred apparently different appearances and eating qualities in the ZN genetic background (Figures 3A–D, 4A–E). ZN‐*Wx* ^*la*^ produced a transparent grain phenotype similar to that associated with ZN‐*Wx* ^*b*^, whereas the grains of ZN‐*Wx* ^*mp*^ were less transparent (Figure 3A–C). The phenotypic values for quality traits and TS for the isoline with ZN‐*Wx* ^*la*^ were intermediate between those of isolines with ZN‐*Wx* ^*b*^ and ZN‐*Wx* ^*mp*^ (Figure 4A–E), suggesting that the *Wx* ^*la*^ allele indeed conferred genetic effects intermediate to those of *Wx* ^*b*^ and *Wx* ^*mp*^.

### Reduced GBSSI activity leads to the *Wx* ^*la*^ phenotype

The *Wx* ^*b*^, *Wx* ^*la*^ and *Wx* ^*mp*^ alleles all have the Int1‐1 mutation; therefore, this mutation should not affect endosperm in the material in this study. Expression analysis revealed that isolines containing ZN‐*Wx* ^*b*^, ZN‐*Wx* ^*la*^, and ZN‐*Wx* ^*mp*^ had similar levels of mature *Wx* mRNA during seed development (Figure S3). However, the activity of *Wx* ^*la*^‐encoded GBSSI was clearly lower than that of *Wx* ^*b*^‐encoded GBSSI (Figure 4F), but higher than that of *Wx* ^*mp*^‐encoded GBSSI. *Wx* ^*la*^ and *Wx* ^*mp*^ each contain a single SNP within the coding sequence compared with *Wx* ^*b*^ (Figure 2C). The structures of the GBSSI proteins translated from *Wx* ^*la*^ and *Wx* ^*mp*^ should be similar, because both possess enzymatic activity. The amino acids produced by the Arg to His mutation in Ex4‐53 did not affect polarity, whereas the Tyr to Ser amino acid substitution caused by the mutation in Ex6‐62 did (Figure 4G–I). Because the effect of the Ex4‐53 mutation was greater than that of the Ex6‐62 mutation, the variation generated by Ex4‐53 should be closer to the active site of the GBSSI protein.

Scanning electron microscopy (SEM) of transverse mature endosperm sections revealed many cavities in the endosperm of ZN‐*Wx* ^*mp*^ and ZN, compared with only a few cavities in that of ZN‐*Wx* ^*b*^ and ZN‐*Wx* ^*la*^ (Figure 3). Therefore, the cavities within the starch granules were probably responsible for the observed differences in the physicochemical properties and transparency between the different genotypes. A reduced AAC is therefore important for the formation of stable, transparent endosperm.

### *Wx* ^*la*^ derives from intragenic recombination

The six mutations in *Wx* generated more than six alleles and some alleles contained two mutations (Figure 2C). Some alleles were formed by a second mutation within an existing allele, such as *wx* and *Wx* ^*mp*^, which contain additional mutations to *Wx* ^*b*^ (Wanchana et al., 2003; Yang et al., 2013). However, *Wx* ^*la*^ contained two existing and widely distributed variations, Int1‐1 and Ex6‐62, and its origin prompted further analysis.

**Figure 5:**
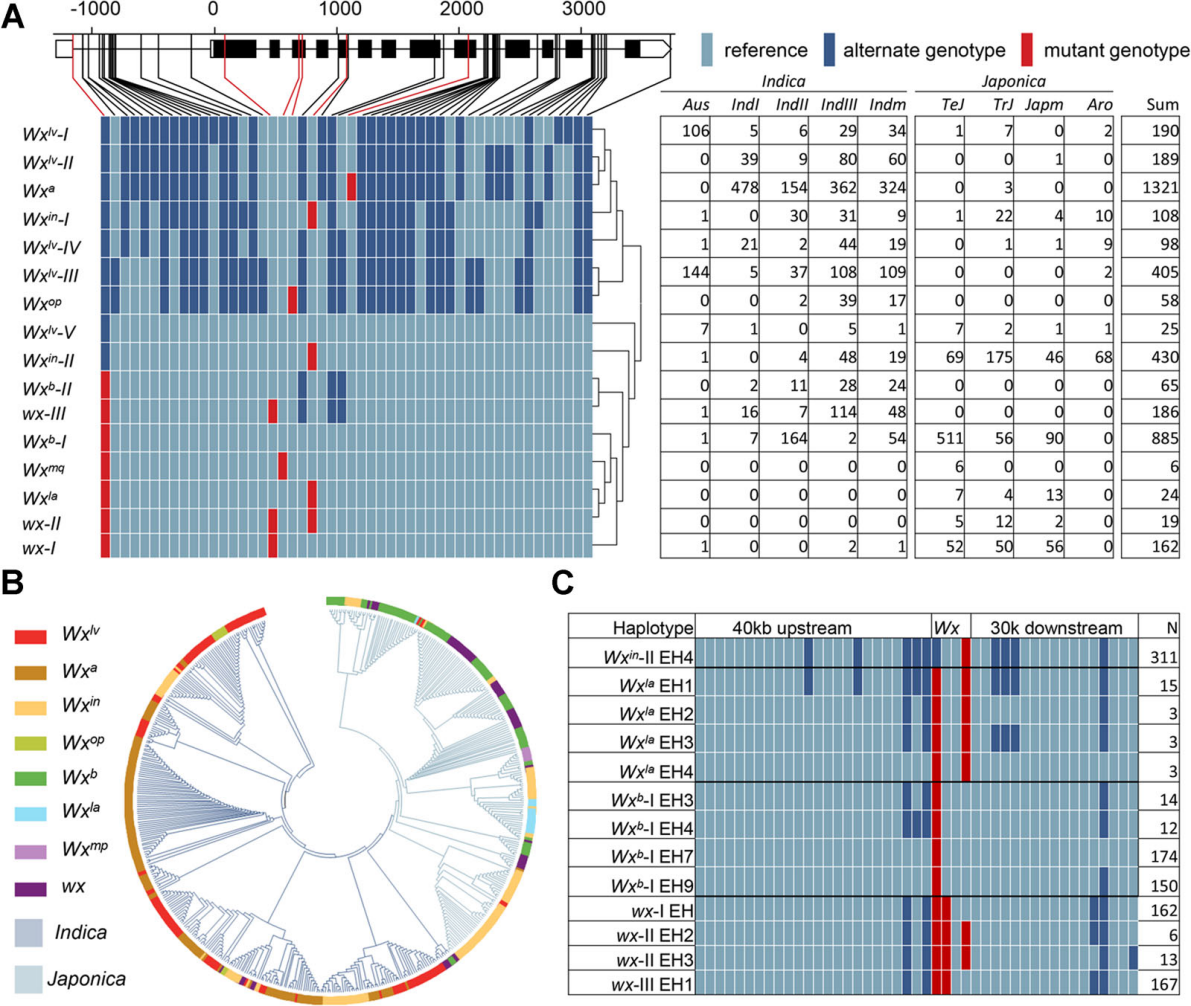
Genetic diversity and evolutionary relationship among multiple *Wx* alleles in rice (**A**) Genotypes and distributions of haplotypes divided by 52 variants within the *Wx* gene. (**B**) Phylogenetic relationship of 200‐kb sequences flanking *Wx* generated from 500 cultivated rice accessions. (**C**) Extended haplotypes of *Wx* ^*in*^, *Wx* ^*la*^, *Wx* ^*b*^, and *wx*.

We performed an intensive haplotype analysis of the *Wx* locus in 4,726 rice accessions (Figures 5A, S4). The *Wx* alleles were divided into 16 haplotypes based on 52 variations; several alleles consisted of one or more haplotype: a single haplotype in *Wx* ^*a*^, *Wx* ^*op*^, *Wx* ^*la*^, *Wx* ^*mp*^, two haplotypes in *Wx* ^*in*^ *Wx* ^*b*^, three haplotypes in *wx*, and five haplotypes in *Wx* ^*lv*^. Among them, *Wx* ^*lv*^‐I, *Wx* ^*lv*^‐II, *Wx* ^*lv*^‐III and *Wx* ^*lv*^‐V, correspond to *Wx* ^*lv*^‐III, *Wx* ^*lv*^‐II, *Wx* ^*lv*^‐IV and *Wx* ^*lv*^‐I, respectively, in the study of Zhang et al. (2019). Clear *indica*–*japonica* differences existed among haplotypes and alleles. *Wx* ^*la*^ was a rare allele and was present only in *japonica* and potentially derived from *Wx* ^*b*^, *Wx* ^*in*^ or *wx*. However, it was not possible to determine the origin of *Wx* ^*la*^ by analyzing variants within the *Wx* locus. Therefore, we studied 100‐kb sequences upstream and downstream of the *Wx* gene to investigate the origin of *Wx* ^*la*^. Phylogenetic analysis of the 200‐kb sequences spanning *Wx* suggested that *Wx* ^*la*^ was most closely related to *Wx* ^*in*^ (Figure 5B).

To determine whether *Wx* ^*la*^ was derived from recombination involving *Wx* ^*in*^, we examined the genomic regions flanking *Wx* in all accessions. In total, 50 extended haplotypes (EH) (Figure S5) were identified, including four extended haplotypes of *Wx* ^*la*^ (Figure 5C). Among these, *Wx* ^*la*^ EH1 differed only in Int1‐1 compared with *Wx* ^*in*^‐II EH4, and *Wx* ^*la*^ EH2 differed only in Ex6‐62 compared with *Wx* ^*b*^‐I EH3. However, the upstream and downstream regions of *Wx* ^*la*^ EH3 derived from different haplotypes. The downstream region of *Wx* ^*la*^ EH3 also derived from *Wx* ^*in*^‐II EH4, whereas the upstream region of *Wx* ^*la*^ EH3 potentially derived from *Wx* ^*b*^‐I EH3, *Wx* ^*b*^‐II EH, *wx*‐I EH, *wx*‐II EH2, *wx*‐II EH3 or *wx*‐III EH1. We observed that existing alleles such as *Wx* ^*op*^ and *Wx* ^*mp*^, which were generated by mutation, possessed a single extended haplotype, whereas alleles such as *Wx* ^*in*^‐II and *wx*‐II, which were generated via recombination, possessed more haplotypes. These findings indicated that *Wx* ^*la*^ was generated from recombination of *Wx* ^*in*^ and *Wx* ^*b*^ or *wx*.

### Intragenic recombination is a major driver of genetic diversity in *Wx*

**Figure 6:**
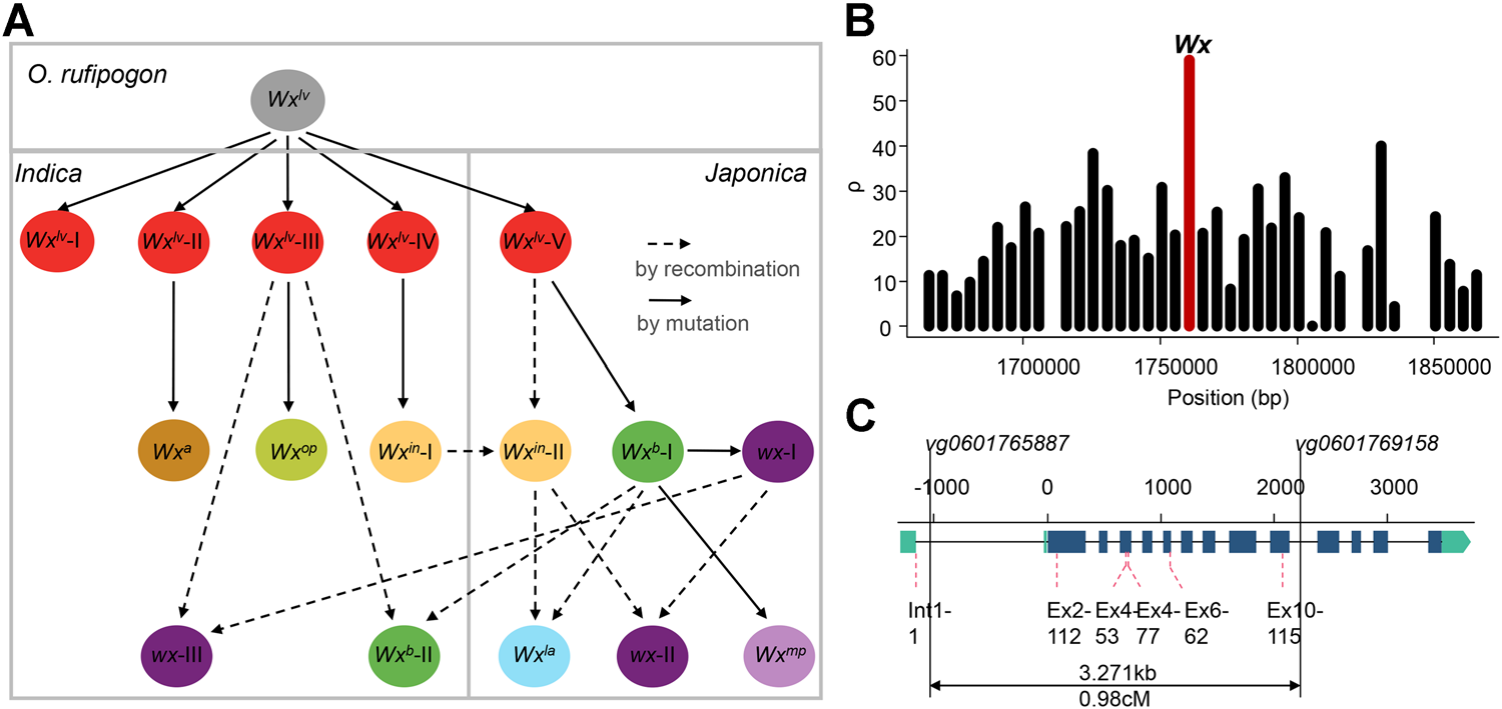
Proposed evolutionary relationships and estimation of the recombination rate at the *Wx* locus (**A**) The evolutionary relationships among *Wx* alleles and haplotypes in rice. (**B**) Estimates of population recombination rate (*ρ*) around the *Wx* locus in 533 cultivars. (**C**) Estimates of the intragenic recombination rate at the *Wx* locus. *vg0601765887* and *vg0601769158* are flanking markers used to screen for recombinants. The location of six functional variations are marked with red dashed lines.

On the basis of the extended haplotypes of *Wx*, we speculated on the potential origins of all *Wx* haplotypes (Figure 6). The *Wx* ^*lv*^ is the ancestral allele and the five haplotypes *Wx* ^*lv*^ *‐I* to *Wx* ^*lv*^ *‐V* all derived from *O. rufipogon*. The *Wx* ^*a*^, *Wx* ^*op*^, *Wx* ^*in*^ *‐I* and *Wx* ^*b*^ *‐I* alleles derived from mutations in *Wx* ^*lv*^ *‐II*, *Wx* ^*lv*^ *‐III*, *Wx* ^*lv*^ *‐IV* and *Wx* ^*lv*^ *‐V*, respectively. The alleles *wx‐I* and *Wx* ^*mp*^ were each derived from *Wx* ^*b*^ by a second mutation; however, the *Wx* ^*in*^‐II, *Wx* ^*b*^‐II, *Wx* ^*la*^, *wx*‐II and *wx‐*III haplotypes were generated by intragenic recombination. These five haplotypes increased the genetic diversity of the *Wx* locus and indicated gene flow between *indica* and *japonica*.

### The *Wx* locus has a high recombination rate

A previous study reported a high intragenic recombination frequency (27.3 kb/cM) at the *Wx* locus (Inukai et al., 2000). We estimated the recombination rate around *Wx* using FastEPRR (Gao et al., 2016) and concluded that the *Wx* locus represented a recombination hotspot (Figure 6B), which greatly contributed to the genetic diversity of the *Wx* gene. The genetic distance between Int1‐1 and Ex6‐62 was estimated to be 0.082 cM, indicating that *Wx* ^*la*^ could easily have resulted from intragenic recombination (Figure S6).

To investigate whether new *Wx* alleles could be generated by intragenic recombination, we crossed Buphopa (allele *Wx* ^*la*^) to Zhenshan97 (allele *Wx* ^*a*^). The *Wx* region flanked by InDel variations *vg0601765887* and *vg0601769158* (http://ricevarmap.ncpgr.cn/) were screened in recombinants among 5,000 F_2_ plants from a cross between Buphopa and Zhenshan97. Forty‐nine recombinants were identified between the markers *wx‐1765887* and *wx‐1769158* (Figure S7; Table S3). Because *vg0601765887* and *vg0601769158* are only 3.271 kb apart, the recombination rate at the *Wx* locus was approximately 3.34 kb/cM, which is much higher than that reported in previous studies. All the recombinants could potentially generate new *Wx* alleles by self‐crossing; therefore, new alleles can be generated by crossing existing accessions containing different alleles.

## DISCUSSION

In this study, we clarified the relationship between taste and traditional quality traits in 533 different cultivated rice varieties. An association study demonstrated that *Wx* was the most important gene that affected taste and eating‐quality traits and a new allele, *Wx* ^*la*^, was identified, which conferred good eating quality and grain transparency. Enzyme activity leading to a low amylose content was the basis for the high quality of the cultivar containing *Wx* ^*la*^ and the *Wx* ^*la*^ allele derived from intragenic recombination. We concluded that the *Wx* gene has undergone complex selection during rice breeding, including intragenic recombination following hybridization, natural mutation, and artificial mutagenesis, and we propose a new evolutionary trajectory for the gene.

Preferences regarding rice quality differ among different cultures; consequently, taste and appearance vary tremendously throughout the world (Calingacion et al., 2014). Natural variation in several major genes accounts for much of the phenotypic variation in appearance and eating quality. The *GS3* and *GW5* genes define grain length and grain width, respectively, whereas *ALK* defines GT (Nakamura et al., 2005; Takano‐Kai et al., 2009; Liu et al., 2017; Zhou et al., 2017). These genes usually possess only two or three functional alleles; however, the *Wx* gene has at least 10 different functional alleles (currently, *Wx* ^*lv*^, *Wx* ^*a*^, *Wx* ^*in*^, *Wx* ^*op*^, *Wx* ^*b*^, *Wx* ^*la*^, *Wx* ^*mq*^, *Wx* ^*mp*^, *Wx* ^*hp*^, and *wx*) (Ayres et al., 1997; Wanchana et al., 2003; Mikami et al., 2008; Liu et al., 2009; Yang et al., 2013; Zhang et al., 2019). The differing and increasing demands for quality has led to the selection of a wide range of mutants of the *Wx* gene, which generally contain a decreased level of amylose. The wild‐type *Wx* allele *Wx* ^*lv*^ first mutated to *Wx* ^*a*^ in *indica* and to *Wx* ^*b*^ in *japonica* (Zhang et al., 2019). Subsequently, *Wx* ^*lv*^ mutated to *wx* ^*in*^ in *indica*, and *Wx* ^*b*^ mutated to *Wx* ^*mq*^ in *japonica* by artificial mutagenesis. However, a low amylose content does not always lead to better taste; for example, *indica* varieties with a higher amylose content than *japonica* are more suitable for making fried rice.

Meiotic recombination is a major driver of genetic diversity, species evolution, and agricultural improvement. Intragenic recombination caused by both crossover and non‐crossover events involving chromatids during meiosis can lead to new alleles or new combinations of existing alleles (Hamant et al., 2006; Mézard et al., 2007). The amount of intragenic recombination in plant genomes varies among species; high recombination frequencies have been observed within genes in maize, but are seldomly observed in *Arabidopsis* (Yao and Schnable, 2005; Smagulova et al., 2011). Recombination events are not evenly distributed across chromosomes, but tend to occur in recombination hotspots. The mean recombination frequency in the rice genome was estimated at one in 250–300 kb/cM, whereas the recombination frequency within the *Wx* locus is about 10 times higher than the mean across the genome (Inukai et al., 2000). In this study, six *Wx* variants (five missense mutations and one nonsense mutation) were identified in a sample of 4,726 varieties. These variations are potentially capable of generating 2^5^ + 1 = 33 different *Wx* alleles. In addition, it is possible to identify new variants that increase the diversity of *Wx* and rice taste. The crossing of varieties containing different *Wx* alleles can generate new alleles through intragenic recombination. After crossing the *Wx* ^*a*^ genotype (Ex10‐115) with the *Wx* ^*la*^ genotype (Int1‐1+Ex6‐62), we identified 49 recombinants (Figure S7) that could generate three types of new alleles: Int1‐1+Ex6‐62+Ex10‐115, Int1‐1+Ex10‐115, and Ex6‐62+Ex10‐115. Such new alleles enrich the genetic diversity of the *Wx* locus and could help to improve rice quality.

## MATERIALS AND METHODS

### Plant materials and phenotyping

Association mapping was performed on a sample of 533 *Oryza sativa* accessions. Information concerning the accessions, including names, countries of origin, geographical locations and subpopulation classification was reported in a previous study (Zhou et al., 2017). Approximately 36 seeds of each accession were germinated and transplanted in an experimental field at Wuhan (N30.49°, E114.36°) for 2 years. Harvested grains of the 533 accessions were air‐dried and stored at room temperature for at least 3 months before testing.

Phenotypic analyses of 10 quality traits were conducted over 2 years. The apparent amylose content (AAC) and gel consistency (GC) of milled rice flours were measured following the method of Bao et al. (2006). Gelatinization temperature (GT) was measured by degree of disintegration of milled rice soaked in KOH solution and evaluated using a 1–7 scale proportionate to the amount of disintegration (Gao et al., 2003). Flour pasting properties were assessed using a rapid visco‐analyzer (Perten, RVA4500) according to the method of Bao et al. (2006). For grain transparency analysis, mature seeds of different *Wx* genotypes were dried in a drying oven (37^o^C) for 24 h to ensure they had the same moisture content. Taste scores of cooked rice were evaluated using a taste analyzer kit (Satake, STA1B‐RHS1A‐RFDM1A, Japan) that included a taste analyzer, a freshness meter, and a hardness and viscosity analyzer. All procedures were carried out according to the manufacturer's protocol.

### Genome‐wide association analyses

Genome‐wide association study (GWAS) analyses of quality traits were separately performed for *indica* and *japonica* accessions and on the entire population, using mixed linear models provided in the EMMAX program (Kang et al., 2010) that also accounted for population structure and relative kinship for statistical association purposes. In each panel, only SNPs with a MAF >5% and missing rates <15% were selected for association analyses. Finally, 5.2 million SNPs were used to estimate population structure and kinship coefficients (Zhao et al., 2015b), and for GWAS. Genome‐wide significance thresholds of the GWAS were determined using a modified Bonferroni correction as described (Li et al., 2012), in which the total number of SNPs (*M*) for threshold calculation was replaced by the effective number of SNPs (*M*e). The calculated genome‐wide significance thresholds, based on a nominal level of 0.05, were *P* = 6.6 × 10^−8^, 8.7 × 10^−8^, and 2.0 × 10^−7^ for the whole population, and *indica* and *japonica* populations, respectively (Table S1). The physical locations of the SNPs were identified using Rice Annotation version 7.0 of variety Nipponbare from Michigan State University (MSU).

### Statistical analyses

Histograms, boxplots, correlations and GWAS analyses were constructed using phenotypic grand means for each variety. The Pearson correlation coefficient *P*‐values were calculated via a two‐sided *t*‐test using the cor.test() function in R (Ihaka and Gentleman, 1996). The phenotypic variation in quality traits explained by multiple SNPs was calculated using the lm() and step() functions in R.

### Transgene analyses

The 7.1‐kb genomic fragments containing the *Wx* gene from Niponbare (*Wx* ^*b*^), Buphopa (*Wx* ^*la*^), Nangeng5055 (*Wx* ^*mp*^) were cloned into the pCAMBIA1301S binary vector (Cambia) digested with *Kpn*I and *Hin*dIII, to generate transgenic complementation constructs. Constructs were first introduced into *E. coli* strain Trans 5α and were sequenced to identify correct clones, which were then introduced into *Agrobacterium tumefaciens* strain EHA105 and transferred into relevant plant material via Agrobacterium‐mediated transformation (Toki, 1997). We used Zhennuo (ZN) as the recipient parent and positive lines were designated ZN‐*Wx* ^*b*^, ZN‐*Wx* ^*la*^, and ZN‐*Wx* ^*mp*^.

### Gene expression and measurement of GBSSI activity

Total RNA was isolated with Trizol reagent (Invitrogen) according to the manufacturer`s instructions. Total RNA was pre‐treated with DNaseI (Invitrogen) and about 2 μg total RNA was used to synthesize first‐strand cDNA using oligo (dT)_18_ primer (Promega). The first‐strand cDNA product was then diluted to a density of 10 ng/μL and 5 μL diluted cDNA was used as a template in a PCR reaction in a total volume of 11 μL. For quantitative real‐time PCR, 5.5 μL of SYBR Green I was added to the reaction mix and PCR was carried out in an ABI QuantStudio6 Flex machine according to the manufacturer's instructions. Melting curves and transcript data were calculated by QuantStudio Real‐Time PCR software. *OsActin1* was used as an internal control and the relative expression level was calculated by 2^−ΔΔ*C*^_t_. Each experiment was performed with at least three replicates.

Activity of GBSSI was measured using a plant Granule‐Bound Starch Synthase ELISA kit (ml076667, Shanghai Enzyme‐Linked Biotechnology Co., Ltd). Immature grains were freshly harvested at 5, 10, 15, 20, and 25 d after flowering. After removing the glumes, husks and embryos, the endosperms were ground into powder in liquid N_2_. The powder was placed in a 1.5‐mL centrifuge tube and analyzed according to the manufacturer's instructions.

### Scanning electron microscopy

Cross‐sections of milled white rice grains were coated with gold under vacuum. Starch granule morphology was examined with a scanning electron microscope (JSM‐6390LV, JEOL, Japan) at an accelerating voltage of 10 kV, a spot size of 30 nm, and at magnifications of 40×, 400×, and 2,500×. Scanning electron microscopy (SEM) analysis was based on at least three biological replications of mounted specimens. All procedures were carried out according to the manufacturer's protocol.

### Haplotype and population genetic analysis

The 4,726 *O. sativa* accessions used in this study consisted of three groups; the first group of 533 accessions was sequenced in our previous study (Zhao et al., 2015a); the second group containing 950 accessions was sequenced by Huang et al. (2011); and the third group consisting of 3,243 accessions was derived from the 3,000 Rice Genomes Project (3KRGP) (Li et al., 2014). The SNP and InDel variation data for *Wx* in all 4,726 accessions are available at RiceVarMap (http://ricevarmap.ncpgr.cn/). Subpopulation identities were inferred using ADMIXTURE (Alexander et al., 2009), and were also queried from RiceVarMap. In total, 101 SNPs and 22 InDels were identified in the 5,036 bp sequence of LOC_Os06g04200. Nucleotide diversity (*π* and *θ*) was calculated using the DnaSP program (Watterson, 1975; Tajima, 1983; Kumar et al., 2016). Haplotypes were extracted using the same program after removing low‐frequency variations and non‐informative InDels. Extended haplotypes (EH) spanning an 80.2‐kb region flanking *Wx* were used to distinguish the origins of *Wx* alleles on the basis of analysis of 4,726 accessions. Haplotypes were constructed from distinct SNPs (frequencies >0.05) identified within the 80‐kb region flanking *Wx*. The phylogenetic tree for 200‐kb sequences spanning *Wx* was constructed using the neighbor‐joining method in Mega 7.0 (Kumar et al., 2016).

### Estimation of recombination rate

The recombination rates around the *Wx* locus were calculated using the R package FastEPRR (Gao et al., 2016). Genotypes of SNPs and InDels in 533 varieties were used to estimate the recombination rate with a 5‐kb sliding window. To estimate the intragenic recombination rate, *indica* accession Zhenshan97 was crossed to *japonica* accession Buphopa. Zhenshan97 and Buphopa carried the *Wx* ^*a*^ and *Wx* ^*la*^ alleles, respectively, at the *Wx* locus. Markers *wx‐1765887* and *wx‐1769158* (Table S3) designed from variations *vg0601765887* and *vg0601769158* (http://ricevarmap.ncpgr.cn) were used to screen for recombinants between *vg0601765887* and *vg0601769158* among 5,000 F_2_.

## CONFLICT OF INTEREST

The authors declare that they have no competing interests.

## AUTHOR CONTRIBUTIONS

H.Z. and D.X. conducted most of the experiments, including phenotyping, association analysis, transformation analysis, and population genetic analysis. D.Z., Y.L., P.L., and B.W. conducted parts of the phenotyping; G.W. and X.L. provided rice germplasm samples; G.G., Q.Z., J.X., S.Y., and X.L. participated in field management and logistics. Y.H. designed and supervised the study. Y.H., H.Z., and D.X. analyzed the data and wrote the paper. All authors reviewed the manuscript and approved the final version of the manuscript.

## Supporting information

Additional Supporting Information may be found online in the supporting information tab for this article: http://onlinelibrary.wiley.com/doi/10.1111/jipb.13011/suppinfo

**Figure S1.** Rapid visco‐analyzer (RVA) pasting viscosity parameters (left) and RVA pasting properties of 533 different rice accessions (right)
BDV, breakdown viscosity; cP, centipoise; CPV, cold paste viscosity; CSV, consistency viscosity; HPV, hot paste viscosity; PKV, peak viscosity; SBV, setback viscosity.
**Figure S2.** Manhattan and QQ plots for 10 quality traits among the entire population of 533 rice accessions
Dashed lines represent the significance thresholds (‐log10(*P*) = 7.18).
**Figure S3.** Expression level of different *Wx* alleles in ZN isolines during endosperm development
ZN is a glutinous rice variety containing the *wx* allele. Plotted values are means ± *SD* (*n* = 5).
**Figure S4.** Distributions and genotypes of *Wx* haplotypes among 52 variants among different subpopulations
The reference genotype is depicted in light gray, alternative genotypes in dark gray, and mutant genotypes in red.
**Figure S5.** Table showing extended haplotypes (EH) corresponding to the 80‐kb region flanking the *Wx* gene (shown as a bar)
The reference genotype is depicted in light blue and alternative genotypes in dark blue.
**Figure S6.** Estimates of genetic distance among six functional variants of the *Wx* locus according to Inukai et al. (2000)
**Figure S7.** The genotypes of recombinants identified from the cross between two accessions carrying *Wx* ^*a*^ and *Wx* ^*la*^ alleles, respectively
**Table S1.** SNPs and candidate genes significantly associated with quality traits
**Table S2.** Genetic effects of six *Wx* variants on apparent amylose content (AAC)
**Table S3.** Primers used in this study
