# Supplementary material for: The origin of Wx la provides new insights into the improvement of grain quality in rice
Source: J Integr Plant Biol. 2021 Mar 26;63(5):878–88. doi: 10.1111/jipb.13011 (PMC8252478; doi:10.1111/jipb.13011)
Supplement: Supplementary file 1 — Figure S1. Rapid visco‐analyzer (RVA) pasting viscosity parameters (left) and RVA pasting properties of 533 different rice accessions (right) BDV, breakdown viscosity; cP, centipoise; CPV, cold paste viscosity; CSV, consistency viscosity; HPV, hot paste viscosity; PKV, peak viscosity; SBV, setback viscosity. Figure S2. Manhattan and QQ plots for 10 quality traits among the entire population of 533 rice accessions Dashed lines represent the significance thresholds (‐log10(P) = 7.18). Figure S3. Expression level of different Wx alleles in ZN isolines during endosperm development ZN is a glutinous rice variety containing the wx allele. Plotted values are means ± SD (n = 5). Figure S4. Distributions and genotypes of Wx haplotypes among 52 variants among different subpopulations The reference genotype is depicted in light gray, alternative genotypes in dark gray, and mutant genotypes in red. Figure S5. Table showing extended haplotypes (EH) corresponding to the 80‐kb region flanking the Wx gene (shown as a bar) The reference genotype is depicted in light blue and alternative genotypes in dark blue. Figure S6. Estimates of genetic distance among six functional variants of the Wx locus according to Inukai et al. (2000) Figure S7. The genotypes of recombinants identified from the cross between two accessions carrying Wx a and Wx la alleles, respectively Table S1. SNPs and candidate genes significantly associated with quality traits Table S2. Genetic effects of six Wx variants on apparent amylose content (AAC) Table S3. Primers used in this study [file JIPB-63-878-s001.docx]

**The origin of *Wx^la^* provides new insights into the improvement of grain quality in rice**

Hao Zhou^†^, Duo Xia^†^, Da Zhao, Yanhua Li, Pingbo Li, Bian Wu, Guanjun Gao, Qinglu Zhang, Gongwei Wang, Jinghua Xiao, Xianghua Li, Sibin Yu, Xingming Lian & Yuqing He*

National Key Laboratory of Crop Genetic Improvement and National Centre of Plant Gene Research (Wuhan), Huazhong Agricultural University, Wuhan, Hubei 430070, China.

^†^These authors contribute equally to these works

*Correspondence should be addressed to H.Y. ([yqhe@mail.hzau.edu.cn](mailto:yqhe@mail.hzau.edu.cn))

**Supplementary data**

**Supplemental Figures: 1-7**

**Supplemental Tables: 1-3**


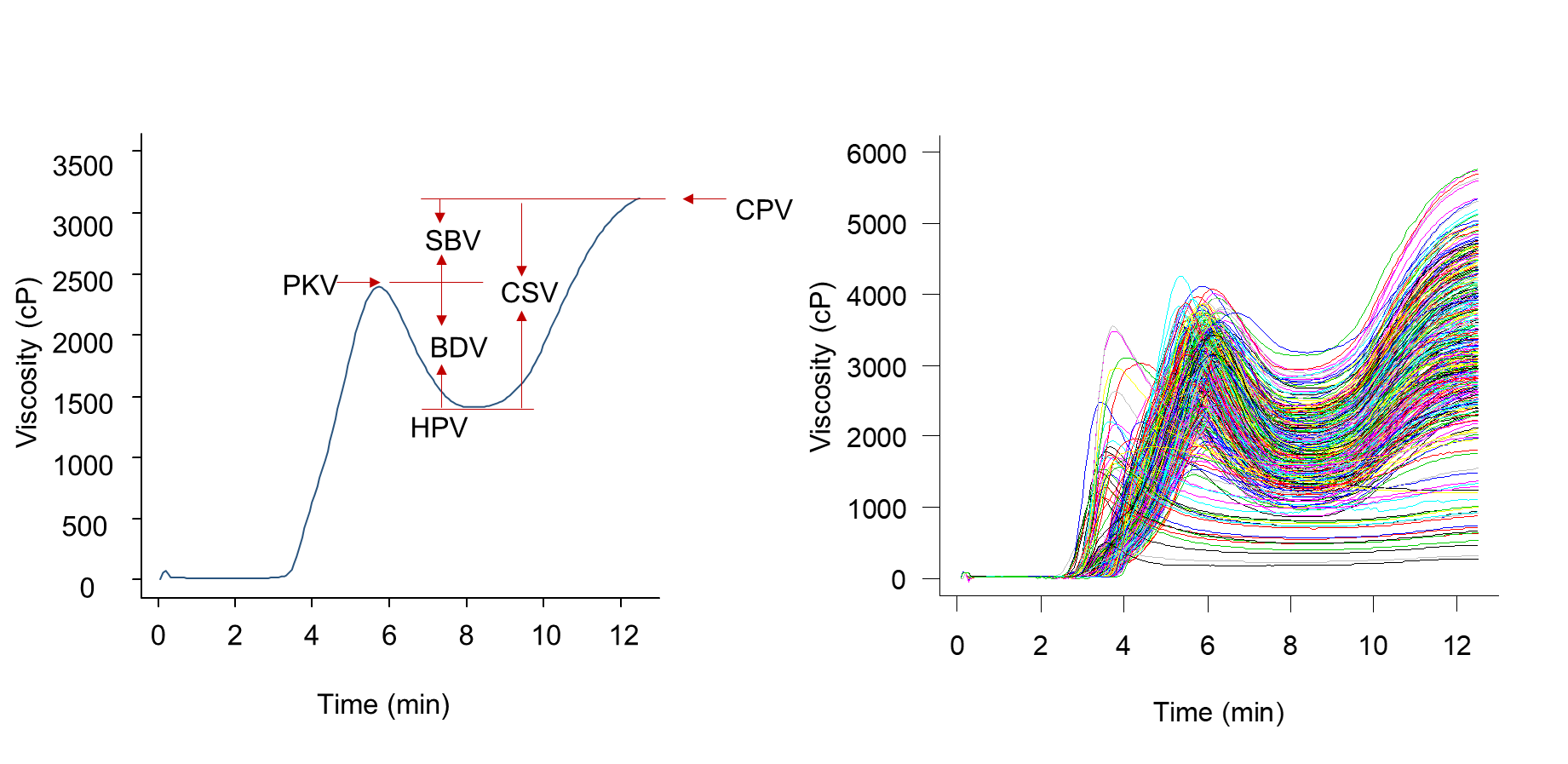


**Figure S1.** Rapid visco-analyzer (RVA) pasting viscosity parameters (left) and RVA pasting properties of 533 different rice accessions (right). Abbreviations: peak viscosity (PKV), hot paste viscosity (HPV), breakdown viscosity (BDV), cold paste viscosity (CPV), setback viscosity (SBV), and consistency viscosity (CSV). cP, centipoise.


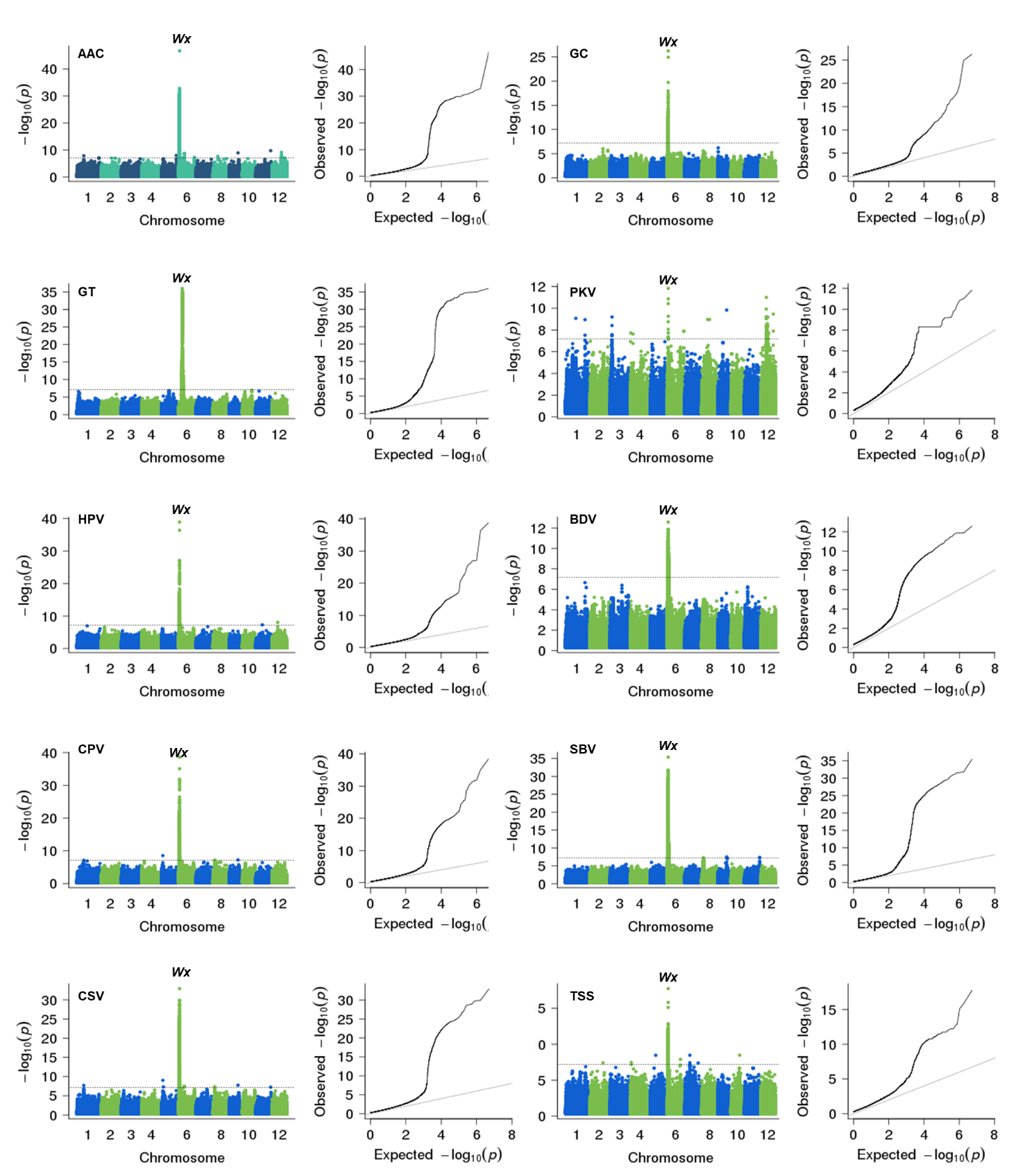


**Figure S2.** Manhattan and QQ plots for 10 quality traits among the entire population of 533 rice accessions. Dashed lines represent the significance thresholds (-log10(*P*) = 7.18).


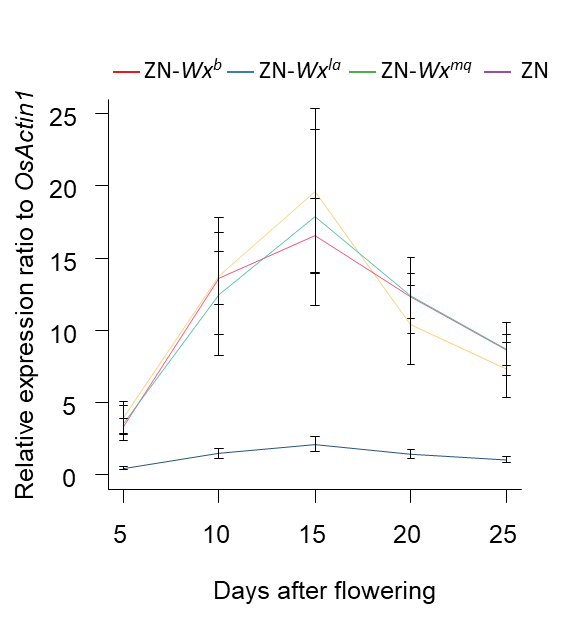


**Figure S3.** Expression level of different *Wx* alleles in ZN isolines during endosperm development. ZN is a glutinous rice variety containing the *wx* allele. Plotted values are means ± SD (*n* = 5).


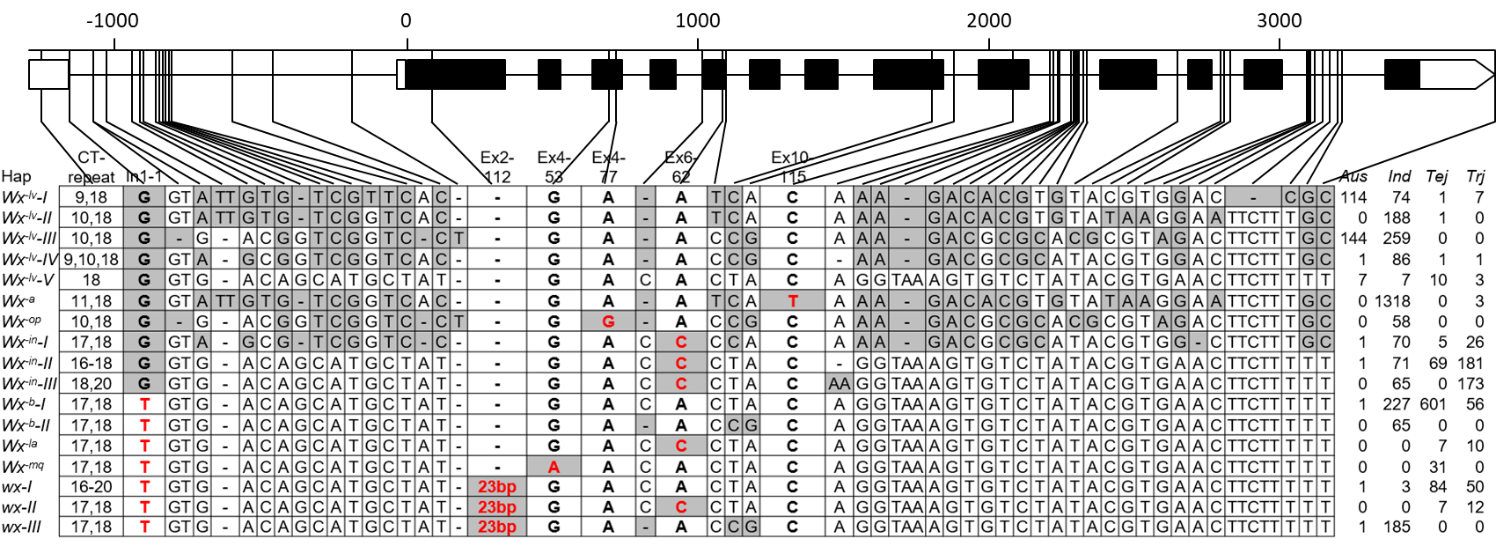


**Figure S4.** Distributions and genotypes of *Wx* haplotypes among 52 variants among different subpopulations. The reference genotype is depicted in light gray, alternative genotypes in dark gray, and mutant genotypes in red.


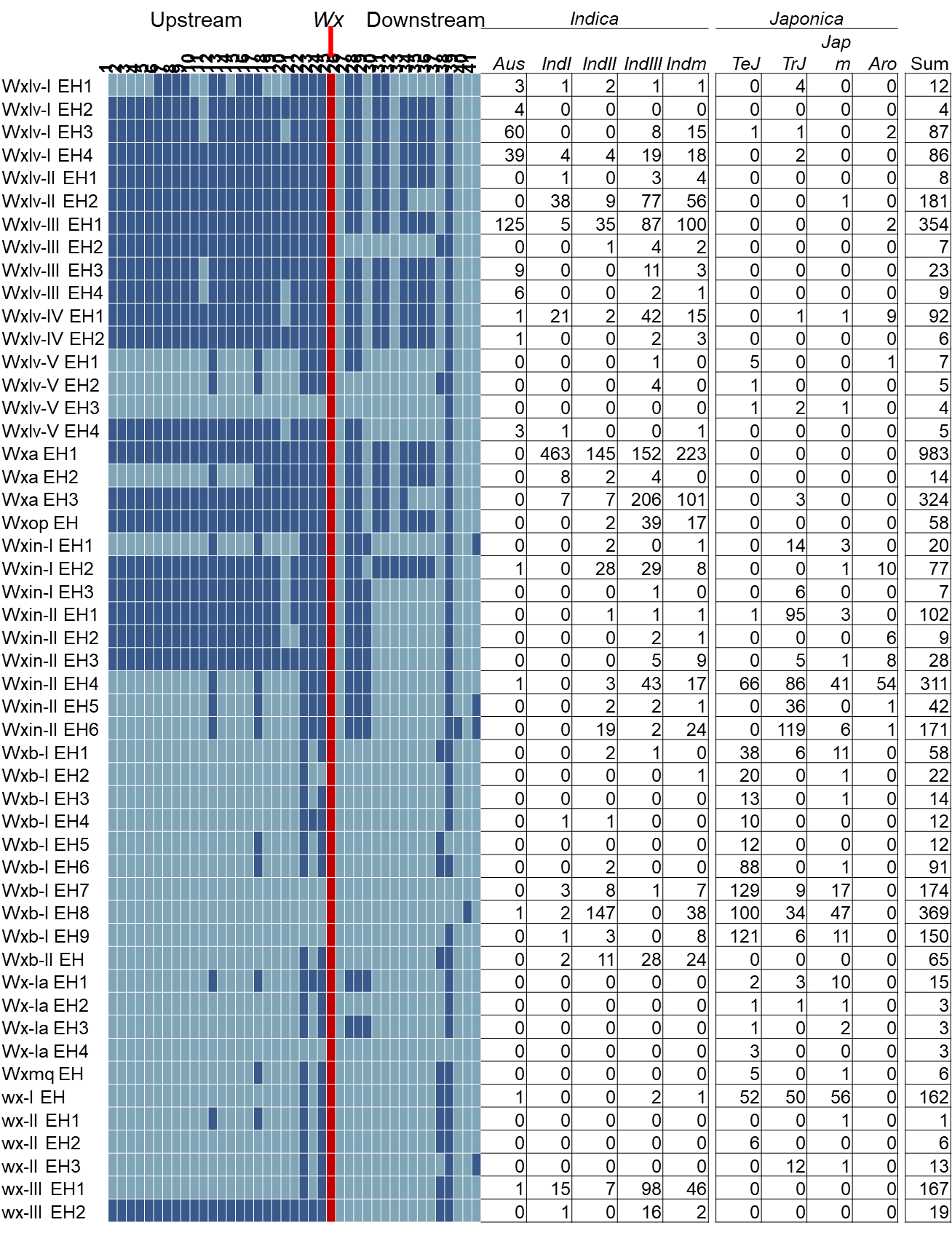


**Figure S5.** Table showing extended haplotypes (EH) corresponding to the 80-kb region flanking the *Wx* gene (shown as a bar). The reference genotype is depicted in light blue and alternative genotypes in dark blue.


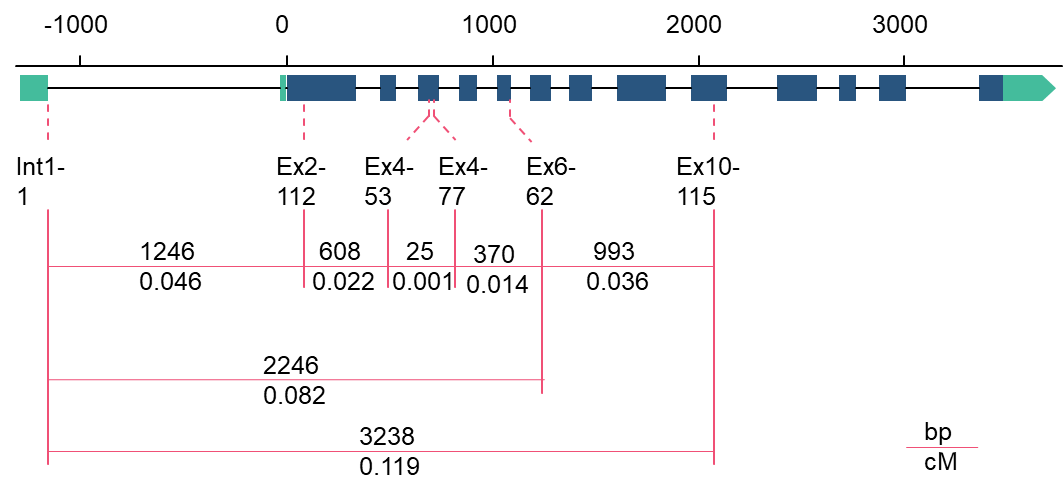


**Figure S6.** Estimates of genetic distance among six functional variants of the *Wx* locus according to Inukai et al. (2000).


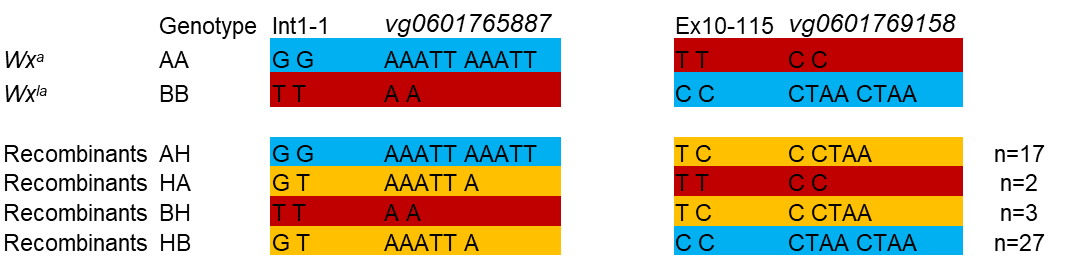


**Figure S7.** The genotypes of recombinants identified from the cross between two accessions carrying *Wx^a^* and *Wx^la^* alleles, respectively.

**Table S1.** SNPs and candidate genes significantly associated with quality traits

| Population | Trait | Alleles | MAF | Chr | Pos | *P*-value | *R^2^* | Dist (kb) | Cand |
| --- | --- | --- | --- | --- | --- | --- | --- | --- | --- |
| All | AAC | T/G | 0.2876 | 6 | 1765761 | 1.84E-47 | 0.6002 | 0 | *Wx* |
| All | GC | T/C | 0.3371 | 6 | 1750498 | 5.40E-27 | 0.3010 | 15 | *Wx* |
| All | GT | G/T | 0.4672 | 6 | 6802721 | 1.02E-36 | 0.3746 | -54 | *ALK* |
| All | PKV | A/C | 0.1533 | 6 | 1826635 | 1.48E-12 | 0.1174 | -61 | *Wx* |
| All | HPV | T/C | 0.3371 | 6 | 1750498 | 1.30E-39 | 0.5102 | 15 | *Wx* |
| All | CPV | T/C | 0.3371 | 6 | 1750498 | 2.56E-39 | 0.521 | 15 | *Wx* |
| All | BDV | T/C | 0.1557 | 6 | 1748967 | 2.63E-13 | 0.2469 | 17 | *Wx* |
| All | SBV | T/G | 0.2876 | 6 | 1765761 | 4.12E-36 | 0.4632 | 0 | *Wx* |
| All | CSV | T/G | 0.2876 | 6 | 1765761 | 1.22E-33 | 0.4418 | 0 | *Wx* |
| All | TSS | T/G | 0.2876 | 6 | 1765761 | 1.76E-18 | 0.3362 | 0 | *Wx* |
| *Indica* | AAC | T/G | 0.2876 | 6 | 1765761 | 7.27E-51 | 0.653 | 0 | *Wx* |
| *Indica* | GC | T/C | 0.3371 | 6 | 1750498 | 5.88E-28 | 0.4236 | 15 | *Wx* |
| *Indica* | GT | A/G | 0.2542 | 6 | 6747298 | 3.07E-55 | 0.7814 | 1 | *ALK* |
| *Indica* | PKV | T/C | 0.3371 | 6 | 1750498 | 9.86E-13 | 0.1102 | 15 | *Wx* |
| *Indica* | HPV | T/C | 0.3371 | 6 | 1750498 | 4.77E-45 | 0.5893 | 15 | *Wx* |
| *Indica* | CPV | T/C | 0.3371 | 6 | 1750498 | 3.71E-49 | 0.6157 | 15 | *Wx* |
| *Indica* | BDV | T/C | 0.4146 | 6 | 1688271 | 4.39E-15 | 0.41399 | 77 | *Wx* |
| *Indica* | SBV | T/C | 0.4737 | 6 | 1816874 | 2.59E-39 | 0.59756 | -51 | *Wx* |
| *Indica* | CSV | G/A | 0.4465 | 6 | 1765976 | 2.58E-40 | 0.49075 | 0 | *Wx* |
| *Indica* | TSS | A/G | 0.4709 | 6 | 1745333 | 8.07E-15 | 0.42982 | 20 | *Wx* |
| *Japonica* | AAC | T/G | 0.2876 | 6 | 1765761 | 1.35E-10 | 0.4149 | 0 | *Wx* |
| *Japonica* | GC | T/C | 0.2566 | 6 | 1719719 | 2.43E-09 | 0.34027 | 46 | *Wx* |
| *Japonica* | HPV | C/A | 0.1265 | 6 | 1886252 | 5.67E-07 | 0.32312 | -121 | *Wx* |
| *Japonica* | CPV | C/A | 0.1265 | 6 | 1886252 | 9.90E-08 | 0.38673 | -121 | *Wx* |
| *Japonica* | BDV | A/G | 0.1734 | 6 | 1394522 | 4.35E-07 | 0.21303 | -349 | *Wx* |
| *Japonica* | SBV | T/C | 0.2566 | 6 | 1719719 | 3.32E-12 | 0.32252 | 46 | *Wx* |
| *Japonica* | CSV | T/G | 0.2876 | 6 | 1765761 | 6.38E-09 | 0.27877 | 0 | *Wx* |

Alleles: Major allele/minor allele. MAF: minor allele frequency. Pos: Position of base pairs for the lead SNP according to the Nipponbare reference genome version 7.0. Dist: Distance from the candidate gene to the peak SNP; Cand: Candidate gene.

**Table S2.** Genetic effects of six *Wx* variants on apparent amylose content (AAC).

|  |  |  |  | **AAC** |  |  |  |
| --- | --- | --- | --- | --- | --- | --- | --- |
| Regression | | Int1 | Ex2 | Ex4-1 | Ex4-2 | Ex6 | Ex10 |
|  | *R^2^* | 60.02% | 44.45% | 1.21% | 0.40% | 0.50% | 23.40% |
|  | *P*-value | 1.97E-91 | 3.67E-59 | 0.02 | 0.17 | 0.61 | 6.90E-28 |
|  |  |  |  |  |  |  |  |
| Multiple regression | |  |  |  |  |  |  |
|  | *R^2^* | Int1 | Ex2 | Ex4-1 | Ex4-2 | Ex6 | Ex10 |
| Forward step | |  |  |  |  |  |  |
| Step 1 | 60.02% | 1.97E-91 |  |  |  |  |  |
| Step 2 | 74.07% | 1.20E-75 | 5.02E-45 |  |  |  |  |
| Step 3 | 74.44% | 5.40E-74 | 5.95E-46 | 1.57E-02 |  |  |  |
| Step 4 | 75.41% | 1.49E-76 | 2.74E-47 | 1.39E-02 | 2.54E-05 |  |  |
| Step 5 | 80.99% | 4.29E-99 | 8.30E-57 | 4.61E-03 | 1.69E-07 | 1.63E-26 |  |
| Step 6 | 80.98% | 1.02E-75 | 1.45E-56 | 4.68E-03 | 1.73E-07 | 2.71E-20 | 7.06E-01 |
| Backward step | |  |  |  |  |  |  |
| Step 7 | 80.99% | 4.29E-99 | 8.30E-57 | 4.61E-03 | 1.69E-07 | 1.63E-26 |  |
| Step 8 | 80.64% | 1.42E-100 | 1.71E-55 |  | 2.11E-07 | 3.92E-26 |  |
| Step 9 | 79.42% | 3.99E-96 | 3.71E-53 |  |  | 4.07E-24 |  |
| Step 10 | 74.07% | 1.20E-75 | 5.02E-45 |  |  |  |  |
| Step 11 | 60.02% | 1.97E-91 |  |  |  |  |  |

**Table S3.** Primers used in this study

| Primers for sequencing Wx | |
| --- | --- |
| SWaxy1F | CCCTCTCTCACCATTCCTTCAGTT |
| SWaxy1R | GGGATTAGAATTTGAAGCTACA |
| SWaxy2F | TCTGCTCAAAGCTCTGTGCATC |
| SWaxy2R | TCACCAGTGGAAGCTAAGCTCA |
| SWaxy3F | GCATATGCGTGATCAGATCATC |
| SWaxy3R | ATGCCGGTGAGCCGCATGAT |
| SWaxy4F | ACGGGTATGAGTAAGATTCT |
| SWaxy4R | GCGCAAGCACAGGGCTGGAG |
| SWaxy5F | GATCCGTGTGTGTTTCAGGG |
| SWaxy5R | CCTGAGTCAAACTACTGCTCCTTC |
|  |  |
| Primers for vector construction | |
| PC-WxPro-F | tacgaattcgagctcggtaccGCAGGTAATTGACACCCCAC |
| WxPro-R | GTGAGAGCCGACATGGTGGTTG |
| WxCDS-F | CAACCACCATGTCGGCTCTCAC |
| PC-WxCDS-R | acgacggccagtgccaagcttTCAGGCTCTTCAAGGAGCAG |
|  |  |
| Primers for real-time PCR of Wx | |
| Wx-RT-F | TCCGAGAGGTTCAGGTCATC |
| Wx-RT-F | ATGAGCTCCTCGGCGTAGTA |
|  |  |
| Primers for recombinant identification | |
| wx-1765887F | TCTGTAAGGTGTTGGGCTGG |
| wx-1765887R | AACCAAACATAACGAACGAAGAT |
| wx-1769158F | CACTTGCACGATATGCCAAA |
| wx-1769158R | CAAAGCCTCACCCCTTCTAA |
